# Reversing the irreversible? A case of successful surgical repair in a late-presenting aortopulmonary window with severe pulmonary hypertension

**DOI:** 10.1186/s43044-026-00726-7

**Published:** 2026-03-09

**Authors:** Rido Mulawarman, Ericko Ongko Joyo, Muhamad Adrin Aefiansyah Putra, Aditya Agita Sembiring, Sisca Natalia Siagian, Oktavia Lilyasari

**Affiliations:** 1 Department Cardiology and Vascular Medicine, National Cardiovascular Centre Harapan Kita, Universitas Indonesia, 11420 Jakarta, Indonesia; 2 Division of Pediatric Cardiology and Congenital Heart Disease, Department of Cardiology and Vascular Medicine, National Cardiovascular Center Harapan Kita, Universitas Indonesia, 11420 Jakarta, Indonesia

**Keywords:** Aortopulmonary window, Pulmonary hypertension, Operability, Adolescent congenital heart disease, Oxygen reactivity, Reversibility

## Abstract

**Background:**

Aortopulmonary window (APW) is a rare congenital heart defect, accounting for only 0.2–0.6% of all congenital cardiac anomalies, and is usually identified and surgically corrected within the first year of life to prevent irreversible pulmonary hypertension (PH). Once pulmonary vascular resistance (PVR) becomes fixed, surgical repair is generally considered contraindicated. However, emerging evidence suggests that pulmonary vascular reactivity may persist in select adolescents, opening a potential therapeutic window beyond infancy.

**Case presentation:**

We report a 15-year-old male with a large APW diagnosed in infancy but lost to follow-up, who presented in adolescence with exertional dyspnea. Initial cardiac catheterization revealed a high indexed PVR (10.89 WU·m²) and absent oxygen vasoreactivity, indicating inoperability. Despite this, preserved biventricular function and lack of cyanosis prompted a trial of targeted medical therapy with sildenafil, spironolactone, lisinopril, and digoxin. After 12 months, repeat catheterization showed dramatic haemodynamic improvement: baseline PVRi decreased to 8.84 WU·m² and fell further to 0.76 WU·m² after 100% oxygen, with a reduction in PVR/SVR ratio from 0.58 to 0.04. The calculated Qp/Qs increased to 16.77, although this extreme value was considered likely overestimated because systemic and pulmonary arterial oxygen saturations were almost identical. Definitive surgical repair was undertaken with excellent early results. On follow-up, the patient remained asymptomatic with preserved biventricular function, low estimated pulmonary artery pressures and no residual APW on echocardiography.

**Conclusion:**

This case suggests that operability in APW may not be irrevocably lost beyond infancy in carefully selected adolescents. It underscores the importance of individualized assessment, medical preconditioning and serial haemodynamic reassessment to unmask latent pulmonary vasoreactivity. Larger series are needed to define which late-presenting patients with APW and PH may safely benefit from definitive surgical repair.

## Learning Points

1. Operability in late-presenting APW is not always lost—Even in adolescents with severe pulmonary hypertension and absent initial vasoreactivity, pulmonary vascular reactivity may persist and can be unmasked with targeted medical therapy and serial reassessment.
2. Medical preconditioning can reverse inoperability—Structured use of pulmonary vasodilators and supportive therapy prior to surgery can significantly reduce pulmonary vascular resistance, allowing safe definitive repair.
3. Dynamic individualized assessment is essential—Single-point hemodynamic measurements may underestimate vascular adaptability; repeated evaluations and multidisciplinary decision-making should guide operability in complex congenital heart disease.

## Background

Aortopulmonary window (APW) is a rare congenital cardiac malformation, comprising only 0.2–0.6% of all congenital heart defects [1]. It is typically identified and surgically repaired during infancy, as untreated lesions rapidly lead to pulmonary overcirculation and irreversible pulmonary vascular remodeling [1, 2]. Once pulmonary hypertension (PH) becomes fixed, particularly in late-presenting adolescents, the window for operability often closes. However, recent evidence suggests that pulmonary vascular resistance (PVR) may remain modifiable in select patients through targeted medical therapy [2, 3]. We report a 15-year-old male with a previously diagnosed but long-neglected aortopulmonary window (APW), who remained clinically stable throughout early childhood despite the presence of a significant shunt. Following a prolonged period of loss to follow-up, he presented in mid-adolescence with progressive exertional symptoms and was initially deemed inoperable due to elevated pulmonary vascular resistance. However, after a structured period of targeted medical therapy, he demonstrated substantial pulmonary vasoreactivity on repeat catheterization, allowing for successful definitive surgical repair. This case supports the notion that, despite haemodynamic profiles initially indicating inoperability, careful medical preconditioning followed by repeated reassessment can reveal a small group of patients who may still be suitable for surgical repair.

## Case illustration

A 15-year-old male was first diagnosed with a large aortopulmonary window (APW) at 6 months of age on transthoracic echocardiography. At that time, he presented with failure to thrive and signs of heart failure. Echocardiography showed an isolated large APW with marked left atrial and left ventricular dilatation and severe functional mitral regurgitation, consistent with LV volume overload. Medical therapy led to clinical improvement; however, because of socioeconomic barriers and inadequate follow-up, the patient was subsequently lost to regular surveillance.

**Fig. 1:**
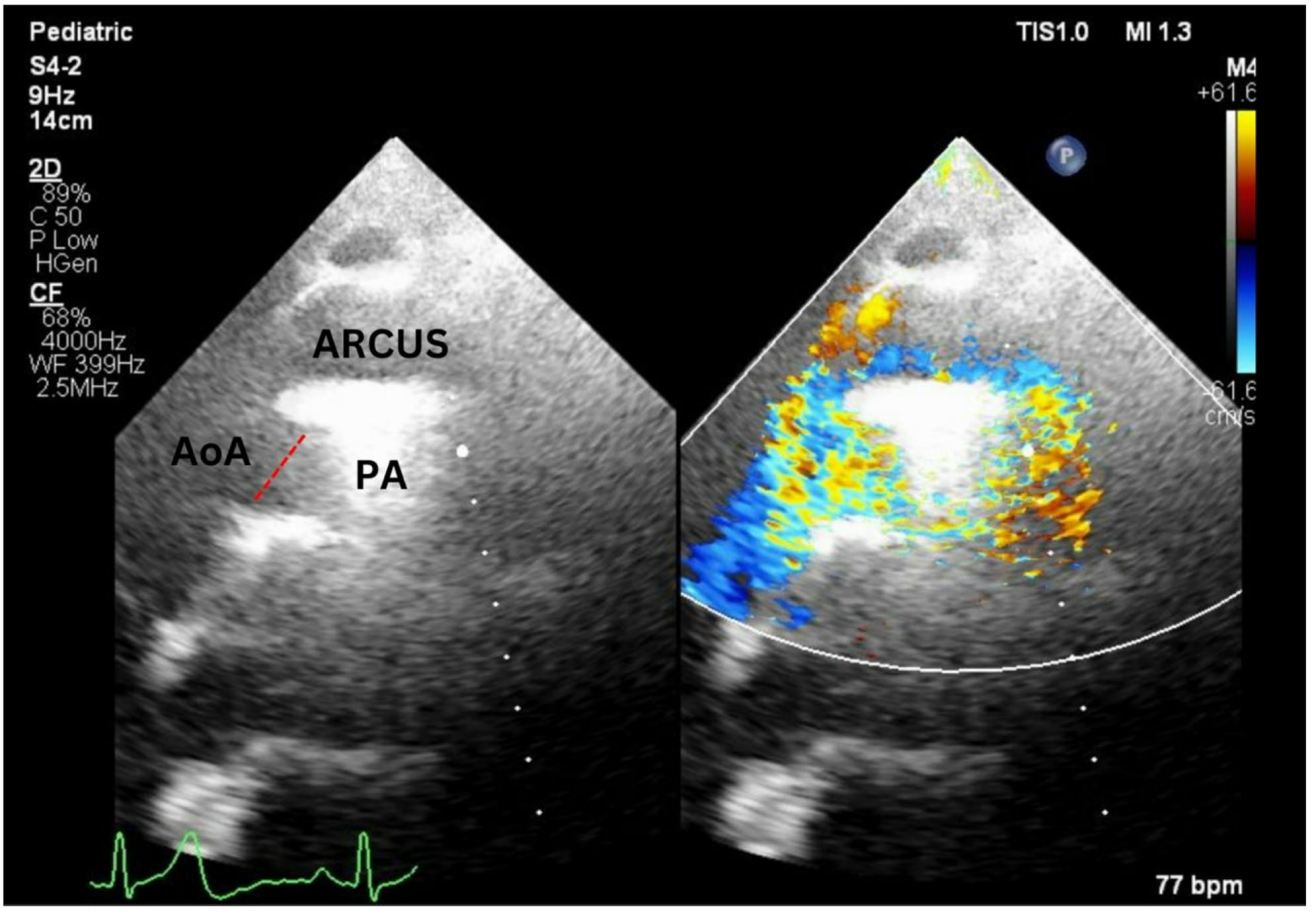
Pre-operative transthoracic echocardiogram demonstrating a large aortopulmonary window (red dashed line) with continuous left-to-right turbulent flow from the ascending aorta (AoA) to the main pulmonary artery (PA) on color Doppler imaging

At the age of 13, the patient developed progressive exertional dyspnoea and reduced exercise tolerance. At the time of re-presentation, he was in NYHA functional class II–III with resting oxygen saturation of 95–97%. Chest radiography demonstrated cardiomegaly with a cardiothoracic ratio of 0.51, a prominent main pulmonary artery and pruning of peripheral pulmonary vessels, without evidence of parenchymal lung disease. Echocardiography revealed a large (approximately 18 mm) bidirectional shunt and turbulent left-to-right flow from the ascending aorta into the main pulmonary artery (Fig. 1). Left ventricular systolic function was preserved (ejection fraction 78%), and right ventricular systolic function was normal (TAPSE 20 mm). There was moderate tricuspid regurgitation with a peak TR jet velocity of 3.65 m/s, corresponding to an RV–RA gradient of 53 mmHg and an estimated systolic pulmonary artery pressure of 60–65 mmHg (assuming right atrial pressure 8–10 mmHg). Additional indices such as LV eccentricity index, right atrial area, RV outflow tract acceleration time and pulmonary regurgitation velocity were not routinely measured and are therefore unavailable.

**Fig. 2:**
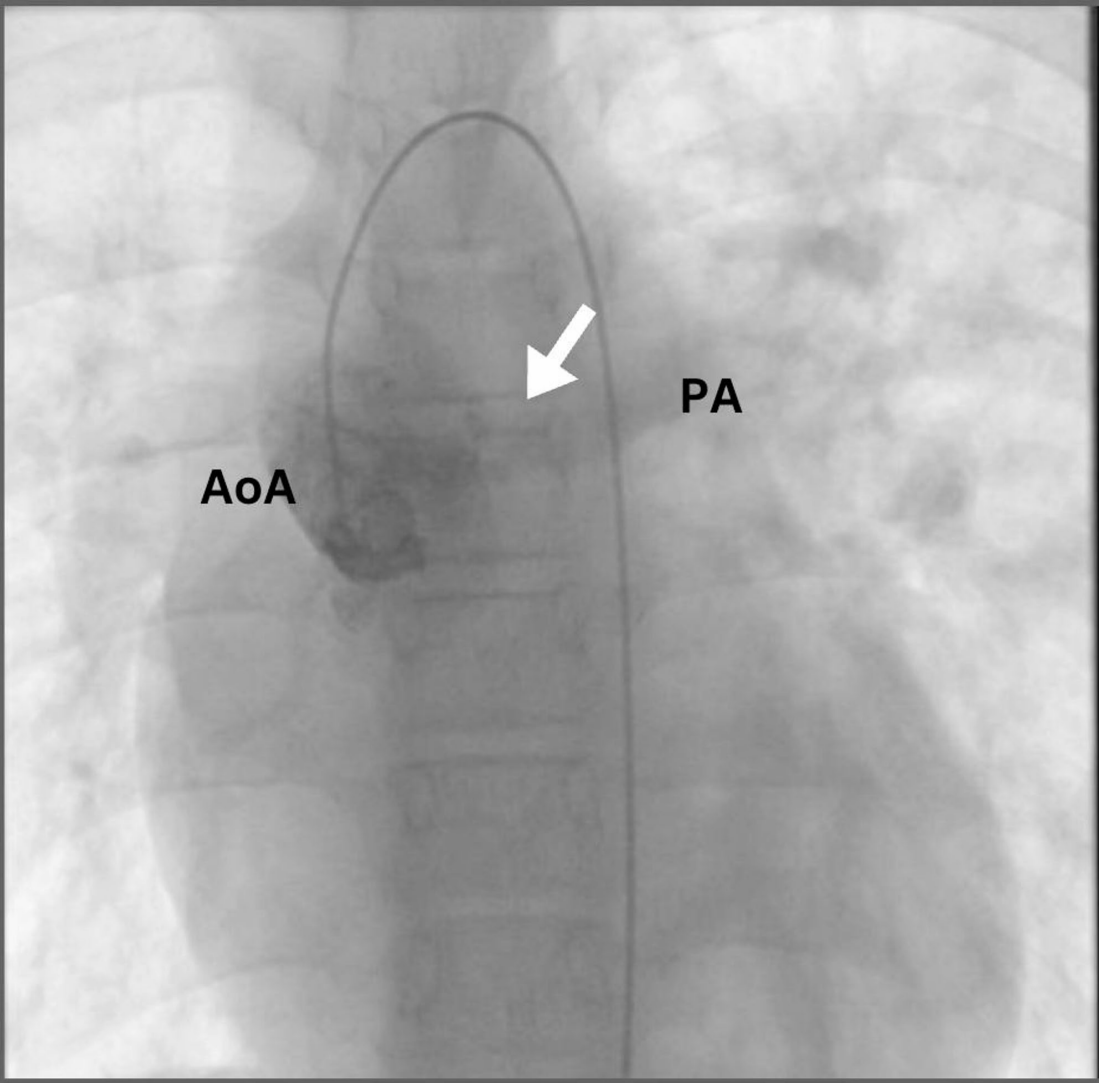
Angiographic image obtained during cardiac catheterization showing contrast opacification of the main pulmonary artery (PA) following ascending aorta (AoA) injection (arrow), consistent with the morphology of an aortopulmonary window

Cardiac catheterization (Fig. 2) was performed and confirmed severe pulmonary hypertension with a high indexed pulmonary vascular resistance (PVRi) and no acute response to 100% oxygen (Table 1). At baseline, the mean pulmonary artery pressure was 78 mmHg with a Qp/Qs of 0.6 and PVRi of 10.89 WU·m². After oxygen challenge, there was no relevant change in mean PA pressure, Qp/Qs or PVRi (10.69 WU·m²), indicating low-flow, high-resistance physiology and a negative vasoreactivity test. Acute testing with inhaled nitric oxide or iloprost was not performed because these agents were not consistently available in our catheterization laboratory. Based on these findings and the markedly elevated wedge pressure (PAWP 41 mmHg), the patient was initially considered inoperable.

**Table 1:** Right-heart catheterization before targeted medical therapy

Parameter	Pre O2 test	Post O2 test
RAP (RA mean pressure), mmHg	14	14
RVP (RV systolic/diastolic), mmHg	101/21	–
PAP (PA systolic/diastolic/mean), mmHg	102/54 (78)	102/55 (77)
PAWP (PCWP mean), mmHg	41	41
Qp/Qs (flow ratio)	0.6	0.6
PVRi (WU·m²)	10.89	10.69
Interpretation	Severe PH, low flow–high resistance	No change after O₂ (negative vasoreactivity)

Despite these findings, several features prompted the multidisciplinary team to reconsider operability such as preserved biventricular systolic function, no resting cyanosis, and no clinical signs of overt right heart failure. A trial of targeted medical therapy was therefore initiated, including sildenafil as a pulmonary vasodilator, spironolactone as a mineralocorticoid receptor antagonist, lisinopril to reduce LV afterload, and digoxin for symptomatic heart failure.

Over the following year, the patient showed gradual clinical improvement. His functional class improved to NYHA I, there were no signs of fluid overload, and resting oxygen saturation remained 95–97%. Repeat echocardiography before reassessment catheterization showed a persistent large APW with bidirectional flow, preserved LV systolic function and normal RV systolic function, with estimated systolic pulmonary artery pressure still in the systemic range.

A second right-heart catheterization after medical optimisation demonstrated marked haemodynamic improvement (Table 2). At baseline, the mean pulmonary artery pressure was 72 mmHg with Qp/Qs 1.5 and PVRi 8.84 WU·m² (PVR/SVR 0.58). After 10 min of 100% oxygen, PVRi fell to 0.76 WU·m² and PVR/SVR to 0.04. The calculated Qp/Qs increased to 16.77. However, systemic and pulmonary arterial oxygen saturations were almost identical, making the denominator of the oximetric formula very small and the Qp/Qs value highly sensitive to minor measurement errors. For this reason, this extreme Qp/Qs was interpreted as an overestimate of the true degree of pulmonary overcirculation, and the focus was placed on the large reduction in PVR and PVR/SVR, which indicated regained operability. Hemodynamic flows and resistances were derived using an oximetry-based indirect Fick method (measured hemoglobin, oxygen saturations, and estimated oxygen consumption). Cardiac output and cardiac index were therefore derived as part of the calculation (indexed to BSA), although cardiac index was not explicitly transcribed in the original cath summary tables. In view of the favourable haemodynamic response and improved clinical status, definitive surgical repair was undertaken. The AP window was closed using a 0.6-mm IMPRA patch. Given the long history of severe PH and previous assessment of inoperability, the surgical team elected to create a small atrial septal defect (approximately 2–3 mm) as a “pop-off” shunt to allow right-to-left decompression in the event of a postoperative pulmonary hypertensive crisis.

**Table 2:** Right-heart catheterization after targeted medical therapy, prior to surgical repair

Parameter	Pre O2	Post O2
RAP (RA mean pressure), mmHg	6	7
RVP (RV systolic/diastolic), mmHg	96/12	93/14
PAP (PA systolic/diastolic/mean), mmHg	98/52 (72)	93/51 (71)
PAWP (PCWP mean), mmHg	Not obtained	Not obtained
LVEDP (surrogate for PAWP), mmHg	12	18
PVR/SVR	0.58	0.04
Qp/Qs (flow ratio)	1.5	16.77
PVRi (WU·m²)	8.84	0.76
Interpretation	Severe PH, low flow–high resistance, reactive to oxygen test	

PAWP/PCWP was not obtained during the March 2025 catheterization; therefore, LVEDP was used as a surrogate for left-sided filling pressure in the calculation of PVR/PVRi. Flows and indexed resistances were derived using an oximetry-based indirect Fick method (Hb, oxygen saturations, and estimated VO₂), with cardiac index derived from calculated cardiac output and BSA (BSA 1.17 m²; Hb 12.9 g/dL; estimated VO₂ 134 mL/min). In an aortopulmonary window, pulmonary artery oxygen saturation represents post-shunt blood and was not used as mixed venous saturation for Qp/Qs calculation; mixed venous saturation was derived from pre-shunt systemic venous sampling (IVC/SVC; proximal to the shunt/RV level)

**Fig. 3:**
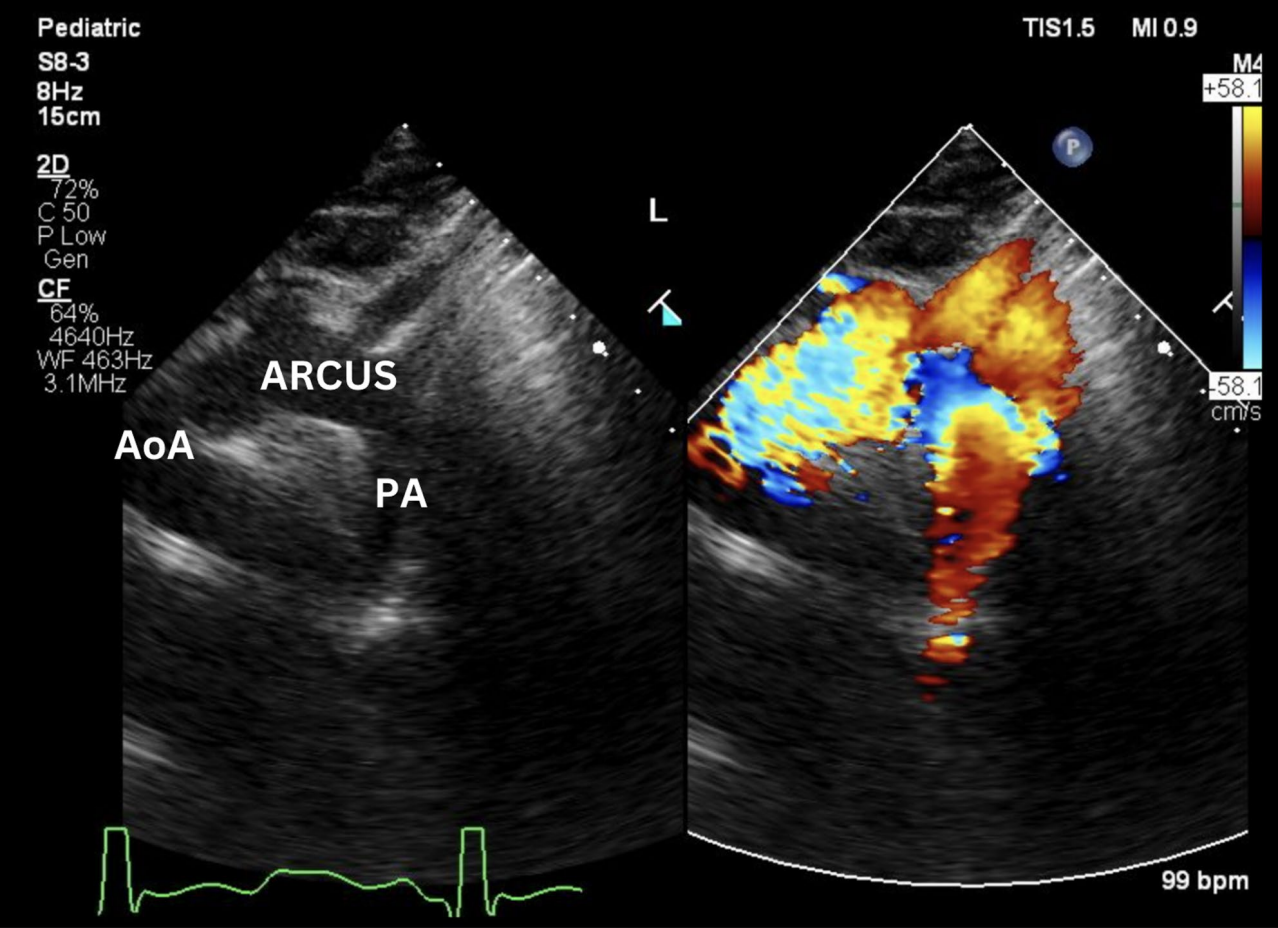
Post-operative suprasternal transthoracic echocardiogram showing complete closure of the aortopulmonary window (APW) between the ascending aorta (AoA) and main pulmonary artery (PA), with no residual flow detected on color Doppler

Postoperative recovery was uneventful. The patient was extubated on postoperative day 2 and discharged home on day 7. A pre-discharge echocardiogram demonstrated complete closure of the AP window, a small residual ASD with minimal left-to-right shunt, preserved LV systolic function (EF 57%), mildly reduced but acceptable RV systolic function (TAPSE 9.9 mm), a very low RV–PA gradient (2 mmHg), and only trivial tricuspid regurgitation. At early outpatient follow-up, echocardiography confirmed durable closure of the AP window (Fig. 3), an ASD of about 2 mm with left-to-right shunt, LV EF 68%, slightly reduced RV function (TAPSE 1.1 cm), mild TR with a TVG of 11 mmHg, low RV–PA gradient (7 mmHg) and trivial pulmonary regurgitation. Clinically, he remained in NYHA class I with oxygen saturation of 97% and no signs of heart failure. Spironolactone and digoxin were discontinued after surgery, while sildenafil and an angiotensin-converting enzyme inhibitor were continued as part of his PH management.

## Discussion

Aortopulmonary window is typically managed with surgical closure in early childhood, before pulmonary vascular disease has progressed to an irreversible stage. Studies have shown that infants who undergo timely correction generally have excellent outcomes, whereas patients who present later often already have advanced pulmonary hypertension and are considered inoperable [2, 4, 5, 7]. On the first catheterization data markedly elevated indexed PVR, high PVR/SVR ratio and no response to 100% oxygen. Our patient would also have been classified in this inoperable category [5, 6].

Operability in congenital heart disease with PH is commonly judged using a combination of clinical findings and haemodynamic thresholds, including PVRi, PVR/SVR and acute vasoreactivity [5–7]. Several proposed decision algorithms regard values around PVRi ≥ 6 WU·m² or PVR/SVR ≥ 0.3 as high-risk and substantially higher values, especially when vasoreactivity is absent, are generally interpreted as arguments against defect closure [5]. However, more recent work has highlighted that these cut-offs do not fully capture the spectrum of pulmonary vascular disease and leave a considerable “grey zone”, especially in children and adolescents [6]. Van der Feen et al. emphasised that a single haemodynamic assessment may not accurately reflect the degree of vascular remodelling or its potential for reversibility, and that treatment and reassessment may be necessary before labelling a patient inoperable [6]. Our patient’s trajectory reflects this approach: although his initial measurements met conventional inoperability criteria, after medical optimisation his haemodynamics fell within a range considered acceptable for surgical repair.

From a mechanistic perspective, this case supports the view that shunt-associated pulmonary vasculopathy develops along a spectrum rather than as a strictly “reversible” or “irreversible” state. Rabinovitch has shown that the early phases are characterised mainly by vasoconstriction, medial thickening and inflammatory changes, which can still respond to targeted therapy, whereas more advanced stages with complex intimal remodelling are much less amenable to regression [4]. Both experimental and clinical data further suggest that interventions acting on the endothelin and nitric oxide pathways may restore a degree of vasoreactivity even in seemingly advanced disease, provided they are introduced before frank Eisenmenger physiology is established [4, 6]. The marked fall in PVRi and PVR/SVR after pre-operative therapy in our case indicates that the pulmonary vascular disease had not yet progressed to an entirely fixed stage.

Several aspects of the clinical profile may help to explain this favourable course. The patient had an isolated, large APW without additional intracardiac defects, and there were no clinical or imaging signs of chronic lung disease or an underlying syndrome. Since childhood and adolescence the patient remained non-cyanotic at rest, and biventricular systolic function was preserved. These characteristics are in line with previous reports suggesting that maintenance of biventricular function and absence of early cyanosis may delay the development of irreversible pulmonary vascular occlusive disease in children with shunt lesions [8, 9]. At the time of the first catheterization, the markedly elevated wedge pressure (PAWP 41 mmHg) indicated a significant post-capillary component in addition to the shunt-related pre-capillary disease. Following medical optimisation, the repeat haemodynamic study was instead dominated by a reversible pre-capillary pattern, with a pronounced reduction in PVR and PVR/SVR in response to oxygen.

The very high post-therapy Qp/Qs ratio of 16.77 requires cautious interpretation. Although the calculations were confirmed, systemic and pulmonary arterial oxygen saturations after 100% oxygen were almost identical, making the denominator of the oximetric formula very small and the resulting ratio extremely sensitive to minor sampling or measurement errors. Such a degree of pulmonary overcirculation would not fit with the patient’s stable NYHA class I status. For these reasons, we did not regard this Qp/Qs as a literal measure of flow, but rather as an indicator of a shift towards predominantly left-to-right shunting. In clinical decision-making, we relied more heavily on the substantial reduction in PVRi and PVR/SVR, the absence of cyanosis, and the concordant improvement in echocardiographic indices of right-sided pressure and function.

Management decisions also reflected the residual perceived risk. Current paediatric PH guidelines advise that patients with borderline haemodynamics should be assessed in specialised centres, and that when doubt persists, decisions should prioritise safety [7]. In our institution, creating a small atrial septal defect as a “pop-off” shunt is an accepted strategy in patients with long-standing or borderline PH undergoing closure of a large systemic-to-pulmonary shunt. In this patient, a 2–3 mm ASD was left intentionally to allow right-to-left decompression should a postoperative pulmonary hypertensive crisis occur, while preserving adequate systemic oxygenation in the early follow-up period.

This case report has important limitations. As a single observation, it cannot be used to redefine operability thresholds or to prove the effectiveness of any particular preconditioning strategy. Several potentially relevant measurements were not available, including cardiac index at both catheterizations, biomarkers such as BNP, more advanced echocardiographic markers of right ventricular–pulmonary artery interaction, and genetic or high-resolution lung imaging to rule out alternative causes of PH. Acute vasoreactivity testing was restricted to 100% oxygen because inhaled nitric oxide and iloprost were not reliably accessible in our catheterization laboratory. In addition, although the haemodynamic and clinical evolution strongly suggest a real reduction in pulmonary vascular resistance, we cannot exclude the presence of residual or recurrent PH over longer-term follow-up.

Despite these limitations, this case adds to the small body of literature suggesting that late-presenting patients with APW and severe PH should not automatically be deemed inoperable on the basis of a single catheterization. In carefully selected adolescents without Eisenmenger physiology, with preserved biventricular function and no alternative cause of PH, structured medical preconditioning followed by serial haemodynamic reassessment may identify a subset in whom definitive repair can be achieved with acceptable risk. Rather than changing existing guidelines, our experience supports a more nuanced, individualised application of current operability criteria that acknowledges the potential for pulmonary vascular plasticity beyond infancy.

## Conclusion

This case illustrates a potential shift in the management of late-presenting aortopulmonary window (APW) complicated by severe pulmonary hypertension. It challenges the long-standing notion that operability is irreversibly lost beyond infancy, emphasizing that pulmonary vascular reactivity can persist in select adolescents despite prolonged exposure to uncorrected left-to-right shunting. Our findings demonstrate that with structured medical preconditioning and serial hemodynamic reassessment, patients initially deemed inoperable may regain candidacy for surgical repair. This underscores the critical need for individualized, dynamic decision-making frameworks in congenital heart disease particularly for rare but potentially reversible lesions like APW.

## Data Availability

No datasets were generated or analysed during the current study.
